# Genome analysis of *Clostridium perfringens* isolates from healthy and necrotic enteritis infected chickens and turkeys

**DOI:** 10.1186/s13104-017-2594-9

**Published:** 2017-07-11

**Authors:** Troels Ronco, Marc Stegger, Kim Lee Ng, Berit Lilje, Ulrike Lyhs, Paal Skytt Andersen, Karl Pedersen

**Affiliations:** 1 National Veterinary Institute, Technical University of Denmark, Bülowsvej 27, 1870 Frederiksberg C, Denmark; 2 Department of Bacteria, Parasites and Fungi, Statens Serum Institut, Artillerivej 5, 2300 Copenhagen S, Denmark; 3 Veterinary Disease Biology, University of Copenhagen, Stigbøjlen 4, 1870 Frederiksberg C, Denmark

**Keywords:** Genome analysis, Necrotic enteritis, Poultry, Virulence factors

## Abstract

**Objective:**

*Clostridium perfringens* causes gastrointestinal diseases in both humans and domestic animals. Type A strains expressing the NetB toxin are the main cause of necrotic enteritis (NE) in chickens, which has remarkable impact on animal welfare and production economy in the international poultry industry. Three pathogenicity loci NELoc-1, -2 and -3 and a collagen adhesion gene *cnaA* have been found to be associated with NE in chickens, whereas the presence of these has not been investigated in diseased turkeys. The purpose was to investigate the virulence associated genome content and the genetic relationship among 30 *C. perfringens* isolates from both healthy and NE infected chickens and turkeys, applying whole-genome sequencing.

**Results:**

NELoc-1, -3, *netB* and *cnaA* were significantly associated with NE isolates from chickens, whereas only NELoc-2 was commonly observed in both diseased turkeys and chickens. A putative collagen adhesion gene that encodes a von Willebrand Factor (vWF) domain was identified in all diseased turkeys and designated as *cnaD*. The phylogenetic analysis based on single nucleotide polymorphisms showed that the isolates generally were not closely related. These results indicate that virulence factors and pathogenicity loci associated with NE in chickens are not important to the same extent in diseased turkeys except for NELoc-2. A putative collagen adhesion gene which potentially could be of importance in regard to the NE pathogenesis in turkeys was identified and need to be further investigated. Thus, the pathogenesis of NE in turkeys appears to be different from that of broiler chickens.

**Electronic supplementary material:**

The online version of this article (doi:10.1186/s13104-017-2594-9) contains supplementary material, which is available to authorized users.

## Introduction

*Clostridium perfringens* is a Gram-positive anaerobic bacterium that causes gastrointestinal diseases in humans and domestic animals [1]. Virulence is primarily due to production of various types of extracellular toxins, and *C. perfringens* strains are assigned a specific toxin type (A–E) dependent on which major toxins (α, β, ε, ι) they produce [2, 3]. Colonization of the intestine by *netB*-positive type A strains is known to be the main cause of necrotic enteritis (NE) in broilers [4, 5], which constitutes a considerable burden to the animal welfare and production yield in the international poultry industry [6]. NetB is essential in the pathogenesis of NE in chickens [4, 5], whereas the prevalence of *netB* is low among diseased turkeys [7, 8]. Few studies of the virulence gene content in strains from turkeys with NE and enteric diseases have been carried out [9] and to our knowledge, there are no reports on whole-genome sequencing of *C. perfringens* isolates from turkeys.

*netB* is found on a 42 kb plasmid-encoded and NE associated pathogenicity locus called NE locus-1 (NELoc-1), which also harbors several other virulence genes [10]. Two other loci have also been found to be associated with NE in chickens, NELoc-2 (11.2 kb) and NELoc-3 (5.6 kb), which are chromosomally and plasmid-encoded respectively [10]. Furthermore, a recent study showed that a collagen adhesion gene, *cnaA* is involved in NE in chickens [11].

The purpose of this study was to investigate the virulence associated genome content and genetic relationship among *C. perfringens* isolates from healthy and NE afflicted chickens and turkeys, applying whole-genome sequencing.

## Main text

### *Clostridium perfringens* isolates

Isolates were sampled from healthy (n = 4) and diseased (n = 13) chickens and from healthy (n = 4) and diseased (n = 9) turkeys. Both chickens and turkeys were from conventionally raised indoor flocks. The samples were collected on 14 different Danish chicken farms between 1997 and 2002 [12], and seven different Finnish turkey farms between 1997 and 2010 [8]. The samples were primarily from the intestine, but six chicken isolates were obtained from liver samples (Additional file 1).

### DNA purification and sequencing

Colonies were grown overnight on Columbia agar base (Oxoid, CM0331, Hampshire, UK) supplemented with 5% calf blood (Statens Serum Institut, Copenhagen, Denmark) at 37 °C under anaerobic conditions (10% CO_2_, 10% H_2_ and 80% N_2_). Single colonies were cultured in 5 ml Trypticase Soy Broth (Becton–Dickinson, Franklin Lakes, N. Jersey,) under same anaerobic conditions, and DNA was purified using the QIAamp DNA Mini Kit (Qiagen, Hilden, Germany), according to the manufacturer’s instructions. Subsequently, DNA libraries were built using the Nextera XT kit (Illumina Inc., San Diego, Ca) according to manufacturer’s instructions, and subjected to whole-genome sequencing using Illumina’s MiSeq platform with paired-end read lengths of 2 × 251 bp.

### Assembly and identification of preselected *C. perfringens* genes

All reads were deposited in the NCBI SRA [13] (Additional file 1) and *de novo* assembled using CLCbio’s Genomics Workbench (GW) v6.5 (CLCbio’s, Aarhus, Denmark) on default settings and a minimum contig-size of 500 NTs. Subsequently, various types of genes were identified using the web-tool MyDbFinder v1.1 [14] with selected verification of open reading frames (ORFs) using the BLASTN implementation in CLCbio’s Main Workbench (MW) v8.5. Nucleotide sequences of various genes were obtained from the Virulence factor database [15] and the NCBI nucleotide database (Additional file 2). All isolates were multi-locus sequence typed (MLST) at PubMLST [16].

### SNP calling and investigation of the general gene content

Single nucleotide polymorphisms (SNPs) were identified using CSI Phylogeny v1.4 [17] on default settings and with *C perfringens* strain ATCC 13124 (NCBI Accession No. NC_008261) as reference chromosome. Thus, all SNPs had a minimum depth of ≥10×, a quality of ≥30 and a distance of ≥10 to the next SNP. The phylogenetic tree was further modified in iTOL v3.1 [18].

Investigation of the general gene content was performed on the assembled contigs using Prokka v1.10 [19] (default settings) and Roary v3.2.4 [20] (–e–mafft settings) for gene detection and annotation, followed by core and accessory genome identification, respectively.

### Statistics

Statistical analyses were carried out using GraphPad Prism v5.02 (GraphPad Software Inc., San Diego, Ca). Differences in the presence of virulence genes and loci between isolates from diseased chickens and turkeys were investigated using a Fisher’s exact test and considered significant when *p* < 0.05.

### Results

Of the 30 isolates, 21 were sequenced to an average coverage of >80× whereas the rest had an average coverage of >40× except isolate T43 that had >30×. The ORFs were generally determined with thresholds of 90% nucleotide identity and 90% coverage of query sequence length. All isolates were confirmed to be of toxin type A as they only carried the *plc* gene (encoding α-toxin) (Table 1) and MLST showed that all isolates were of unknown sequence type (STs), except two isolates (C26 and C31) from diseased chickens that were of ST21.

**Table 1:** Virulence genes and loci identified in 30 *C. perfringens* isolates

Isolate	State	Type	*cnaD*	*cnaA*	*netB*	NELoc-1	NELoc-2	NELoc-3
C1	H	A	+			0	0	0
C3	H	A				12	18	20
C7	H	A	+			3	*100*	20
C8	H	A				9	*100*	20
C24	D	A		+	+	*97*	*100*	*100*
C25	D	A		+		21	*100*	*100*
C26	D	A		+	+	*100*	*100*	*100*
C27	D	A	+			0	0	0
C31	D	A		+	+	*100*	*100*	*100*
C32	D	A	+			18	0	20
C33	D	A	+	+	+	*88*	*100*	*80*
C36	D	A		+	+	*100*	*100*	*100*
C37	D	A			+	*100*	*100*	60
C41	D	A			+	*100*	*100*	*100*
C48	D	A	+	+	+	*94*	*100*	*100*
C124	D	A	+	+	+	*100*	*100*	*80*
C125	D	A			+	*100*	*100*	*100*
T1	D	A	+			0	*100*	0
T5	D	A	+			27	*100*	20
T6	D	A	+			27	0	40
T11	D	A	+	+	+	*100*	*100*	*100*
T14	D	A	+			27	*100*	40
T16	D	A	+			9	0	20
T46	D	A	+			12	*100*	40
T53	D	A	+		+	*100*	0	40
T84	D	A	+			12	*100*	40
T18	H	A	+			21	0	60
T22	H	A		+		9	0	40
T34	H	A				9	0	20
T43	H	A	+			12	*100*	20

The + mark indicates gene presence in isolates from healthy (H) or diseased (D) chickens (C) and turkeys (T). The prevalence (in %) of NELoc-1, -2 and -3 genes are shown. High prevalence of genes is in italics

*netB* was only found in isolates from diseased poultry and primarily in chickens. Of the NE isolates from chickens, 77% (10/13) were *netB*-positive, whereas only 22% (2/9) of the NE isolates from turkeys carried *netB* (*p* = 0.0274) (Table 1). The NELoc-1 and -3 associated genes (Additional file 3), were primarily observed among isolates from diseased chickens (Table 1). The isolates could be divided into two types of groups. A high prevalence group (HPG) that on average carried 94% of the NELoc-1 genes, and a low prevalence group (LPG) that on average carried 10% of the genes (Table 1). As with NELoc-1, the isolates were divided into a HPG that on average carried 96% of the NELoc-3 genes, and a LPG that on average carried 24% of the NELoc-3 genes (Table 1). The NELoc-1 HPG included 77% (10/13) of NE isolates from chickens and 22% (2/9) of the NE isolates from turkeys (*p* = 0.0274). The NELoc-3 HPGs had 77% (10/13) of NE isolates from chickens and in a single isolate (1/9) from a diseased turkeys (*p* = 0.0075). In contrast to the NELoc-1 and -3 genes, the NELoc-2 genes (Additional file 4), when identified in an isolate, were all conserved except in isolate C3 that carried 18% of the genes (Table 1). NELoc-2 was found in 85% (11/13) of the NE isolates from chickens, and in 67% (6/9) of the NE isolates from turkeys (*p* = 0.6090). Only two isolates from healthy chickens and a single isolate from a healthy turkey carried NELoc-2 (Table 1). Sixty-two % (8/13) of the isolates from diseased chickens carried *cnaA* whereas this gene was found only in one diseased turkey (*p* = 0.0306). A single healthy turkey carried *cnaA* (Table 1).

**Fig. 1:**
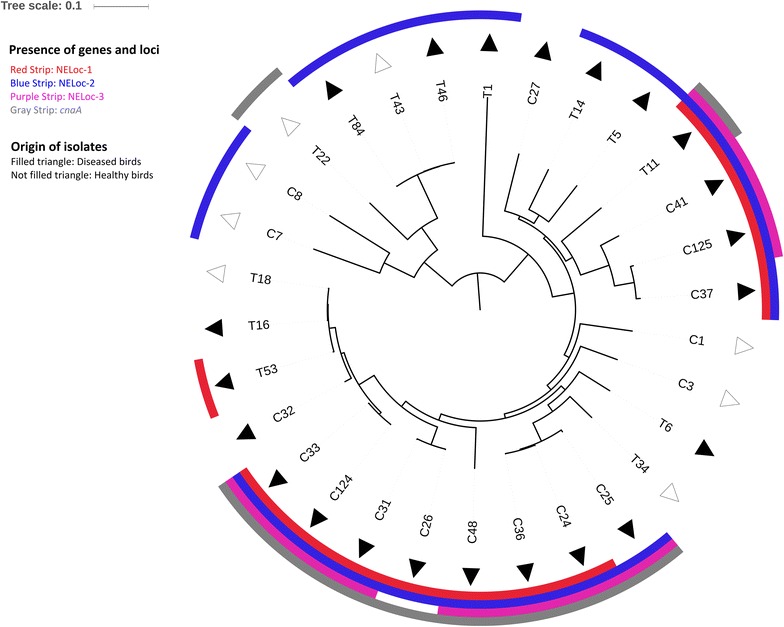
Phylogenetic tree including the distribution of important virulence genes and loci. The maximum likelihood approximated tree based on 50,643 SNPs, shows that 30 isolates from healthy and diseased chickens (C) and the turkeys (T) did not cluster into representative groups. NELoc-1, -3 and *cnaA* were significantly present in NE isolates from chickens, whereas NELoc-2 was associated with NE isolates from both chickens and turkeys. Reference strain *C perfringens* ATCC 13124 is not included. The *scale* represents substitutions per site

The SNP analysis included 50,643 variant positions and 61.9% of the reference genome was covered by all isolates. A phylogenetic analysis revealed no specific clustering among the poultry isolates which in overall were not closely related (Fig. 1). The investigation of the general gene content showed a putative collagen adhesion gene of 2787 nucleotides (NTs) (Additional file 5) here labelled *cnaD*, which was present in all isolates from diseased turkeys (9/9) but only in 39% (5/13) of diseased chickens (*p* = 0.0055). In healthy birds, *cnaD* was found in half (2/4) of both the turkeys and chickens (Table 1). The *cnaD* gene encodes a 928 long amino acid (AA) sequence (Additional file 5), which was analyzed using BLASTP v2.6.1 on default settings [21]. The best hit was an identical *C. perfringens* protein of 928 AAs (NCBI Accession No. WP_011590364) with a query coverage of 100% and an AA identity of 100%. This protein contained two types of conserved domains. A single von Willebrand Factor (vWF) type A domain of 142 AAs (NCBI accession no. smart00327) and three Cna protein B-type domains each of 63 AAs (NCBI accession no. pfam05738).

### Discussion

In this study we carried out genome analysis of *C. perfringens* isolates from healthy and NE afflicted turkeys and chickens. To our knowledge it is the first time *C. perfringens* isolates from turkeys have been whole-genome sequenced and made publicly available. The majority of the all isolates were found to be of unknown ST. This is probably because no *C. perfringens* isolates from the poultry environments investigated in this study, have previously been MLST and thus there are currently no defined MLST schemes that fits these isolates. A phylogenetic analysis confirmed that the majority of isolates were not closely related but constituted a relative diverse population of different genetic backgrounds.

NELoc-1 that carries *netB*, was found only in isolates from poultry with NE and primarily in chickens where 77% of the NE isolates were *netB*-positive (Table 1). Previous studies of chicken isolates showed that *netB* was predominantly present (>80%) among the NE isolates, but absent or rarely detected (<4%) in healthy chickens [4, 5]. Few studies of virulence genes in isolates from turkeys have been carried out. The turkey isolates analyzed in this study were from a previous study [8] where *netB* was found in 26% (14/55) of the NE isolates from turkeys, whereas all isolates from healthy turkeys were *netB*-negative. In another study [7], *netB* was not found in 42 NE isolates from turkeys. Like *netB*, *cnaA* was significantly present among NE isolates from chickens compared to a single NE isolate from a turkey (Table 1). Wade et al. [11] found *cnaA* only in diseased chickens whereas another study [22] also identified *cnaA* in isolates from healthy chickens. Additionally, NELoc-2 and -3 were also primarily found in isolates from diseased chickens. NELoc-1, -2 and -3 were initially discovered in broilers with NE [10] which is why they also in this study were found highly represented among diseased chickens. In contrast, only NELoc-2 was present in the majority of the NE isolates from turkeys, suggesting that this pathogenicity locus may be associated with NE pathogenesis in turkeys (Fig. 1).

Interestingly, a putative collagen adhesion gene was discovered in all diseased turkeys and according to a suggested lettering system presented by Wade et al. [11], it has here been designated as *cnaD*. This gene encodes a vWF type A domain and three Cna protein B-type domains. These two types of domains have been shown to be involved in collagen binding [23]. A single Cna protein B-type domain, identical to those found in this study, is encoded by *cnaA* [11] and the vWF type A domain is also involved in collagen binding [24]. Thus, it may be suggested that these adhesion properties play an important role when *C. perfringens* strains attach to collagen in the intestine of turkeys and cause NE. This should be further investigated via in vitro studies. In a study of turkeys by Saita et al. [9], it was indicated that *C. perfringens* was not only associated with NE showing the same clinical and pathological changes as in broiler chickens, but also with other manifestations of intestinal disorders. These observations suggest that both the pathological manifestations and the pathogenesis of NE are different between turkeys and chickens.

To summarize, NELoc-1, -3, *netB* and *cnaA* were significantly associated with NE isolates from chickens, whereas only NELoc-2 was associated with NE isolates from both turkeys and chickens. Thus, *C. perfringens* virulence genes and loci in chickens with NE do not seem to be important to the same extent in diseased turkeys, suggesting that the NE pathogenesis is different in these two avian species. A putative collagen adhesion gene, *cnaD* was identified in all diseased turkeys and could potentially be of importance in regard to the NE pathogenesis.

## Limitations

An increased sample size would have provided a more robust foundation for these findings. The expression of virulence genes could have been investigated through RNA sequencing which potentially could have further elucidated their importance during the NE pathogenesis. It should be mentioned that NE is a multifactorial disease and predisposing factors like feed composition, mycotoxins, temperature and hygiene stress do also play a considerable role during development of disease outbreaks on poultry farms [5, 25, 26]. Thus, it is not only the genetic profile of virulent *C. perfringens* strains that dictate the pathogenesis of NE.

## Additional files

**Additional file 1: Table S1.** Thirty C. *perfringens* isolates from healthy and NE infected poultry. The pdf-file contains background information of each isolate including SRA accession numbers.

**Additional file 2: Table S2.** Descriptions of various *C. perfringens* genes. The pdf-file contains description of gene products, NCBI accession numbers and references of various *C. perfringens* genes.

**Additional file 3: Table S3.** Presence of NELoc-1 and -3 genes in the high prevalence groups. In this pdf-file, information regarding absence/presence of the ORFs that constitute NELoc-1 and -3 can be found. The table includes locus tags and gene product descriptions.

**Additional file 4: Table S4.** NELoc-2 genes identified among the *C. perfringens* isolates. In this pdf-file, information regarding absence/presence of the ORFs that constitute NELoc-2 can be found. The table includes locus tags and gene product descriptions.

**Additional file 5.** NT and AA sequence of the putative collagen adhesion gene *cnaD.*

## Abbreviations

AA: amino acid
GW: Genomics Workbench
HPG: high prevalence group
LPG: low prevalence group
MW: Main Workbench
MLST: multi locus sequence typing
NE: necrotic enteritis
NELoc: necrotic enteritis locus
NT: nucleotide
ORF: open reading frame
ST: sequence type
vWF: von Willebrand Factor
