# Supplementary material for: Genome analysis of Clostridium perfringens isolates from healthy and necrotic enteritis infected chickens and turkeys
Source: BMC Res Notes. 2017 Jul 11;10:270. doi: 10.1186/s13104-017-2594-9 (PMC5504799; doi:10.1186/s13104-017-2594-9)
Supplement: Supplementary file 1 — Additional file 1: Table S1. Thirty C. perfringens isolates from healthy and NE infected poultry. The pdf-file contains background information of each isolate including SRA accession numbers. [file 13104_2017_2594_MOESM1_ESM.pdf]

Table S1. 30 C. *perfringens* isolates from healthy and NE infected poultry

Isolate	Animal	State	Sample	Farm	Country/year	Reference	SRA acc. no.
C1	Chicken	Healthy	Intestine	1	Denmark/2001	(Nauerby et al., 2003)	SRR4448030
C3	Chicken	Healthy	Intestine	1	Denmark/2001	(Nauerby et al., 2003)	SRR4448031
C7	Chicken	Healthy	Intestine	2	Denmark/2001	(Nauerby et al., 2003)	SRR4448028
C8	Chicken	Healthy	Intestine	2	Denmark/2001	(Nauerby et al., 2003)	SRR4448029
C24	Chicken	Diseased	Intestine	3	Denmark/2001	(Nauerby et al., 2003)	SRR4457405
C25	Chicken	Diseased	Intestine	4	Denmark/2000	(Nauerby et al., 2003)	SRR4457404
C26	Chicken	Diseased	Intestine	5	Denmark/2000	(Nauerby et al., 2003)	SRR4457403
C27	Chicken	Diseased	Liver	6	Denmark/1997	(Nauerby et al., 2003)	SRR4457402
C31	Chicken	Diseased	Liver	7	Denmark/1998	(Nauerby et al., 2003)	SRR4457406
C32	Chicken	Diseased	Liver	8	Denmark/1998	(Nauerby et al., 2003)	SRR4457397
C33	Chicken	Diseased	Liver	9	Denmark/1998	(Nauerby et al., 2003)	SRR4448032
C36	Chicken	Diseased	Liver	10	Denmark/1998	(Nauerby et al., 2003)	SRR4457399
C37	Chicken	Diseased	Intestine	11	Denmark/1999	(Nauerby et al., 2003)	SRR4457398
C41	Chicken	Diseased	Intestine	10	Denmark/2002	(Nauerby et al., 2003)	SRR4457408
C48	Chicken	Diseased	Intestine	12	Denmark/2002	(Nauerby et al., 2003)	SRR4457407
C124	Chicken	Diseased	Liver	13	Denmark/1997	(Nauerby et al., 2003)	SRR4457401
C125	Chicken	Diseased	Intestine	14	Denmark/2000	(Nauerby et al., 2003)	SRR4457400
T1	Turkey	Diseased	Intestine	15	Finland/1998	(Lyhs et al., 2013)	SRR4434753
T5	Turkey	Diseased	Intestine	16	Finland/2005	(Lyhs et al., 2013)	SRR4434754
T6	Turkey	Diseased	Intestine	17	Finland/2005	(Lyhs et al., 2013)	SRR4434751
T11	Turkey	Diseased	Intestine	16	Finland/2006	(Lyhs et al., 2013)	SRR4434752
T14	Turkey	Diseased	Intestine	18	Finland/2006	(Lyhs et al., 2013)	SRR4434749
T16	Turkey	Diseased	Intestine	17	Finland/2008	(Lyhs et al., 2013)	SRR4434750
T18	Turkey	Healthy	Intestine	17	Finland/2009	(Lyhs et al., 2013)	SRR4434747
T22	Turkey	Healthy	Intestine	19	Finland/2009	(Lyhs et al., 2013)	SRR4434748
T34	Turkey	Healthy	Intestine	17	Finland/2009	(Lyhs et al., 2013)	SRR4434755
T43	Turkey	Healthy	Intestine	20	Finland/2009	(Lyhs et al., 2013)	SRR4434756
T46	Turkey	Diseased	Intestine	18	Finland/2010	(Lyhs et al., 2013)	SRR4434757
T53	Turkey	Diseased	Intestine	21	Finland/2010	(Lyhs et al., 2013)	SRR4434758
T84	Turkey	Diseased	Intestine	16	Finland/2011	(Lyhs et al., 2013)	SRR4434759
