# Supplementary material for: Genome analysis of Clostridium perfringens isolates from healthy and necrotic enteritis infected chickens and turkeys
Source: BMC Res Notes. 2017 Jul 11;10:270. doi: 10.1186/s13104-017-2594-9 (PMC5504799; doi:10.1186/s13104-017-2594-9)
Supplement: Supplementary file 2 — Additional file 2: Table S2. Descriptions of various C. perfringens genes. The pdf-file contains description of gene products, NCBI accession numbers and references of various C. perfringens genes. [file 13104_2017_2594_MOESM2_ESM.pdf]

Table S2 Description of various *C. perfringens* genes

Gene	Location	Product	Activity	NCBI acc. no.	Reference
plc	chromosome	Alpha (α) toxin	Phosphlipase C	KP143661	-
cpb	plasmid	Beta (β) toxin	β pore-forming	KP064409	[1]
**exp**	plasmid	Epsilon (ε) toxin	β pore-forming	JX010451	[2]
iap	plasmid	Iota (ι) toxin	Actin ADP-ribosylation	NC_015712	[3]
cnaA	chromosome	Collagen adhesin	Adherence	KT749987	[4]
**NELoc-1 33 ORFs** Incl**.** *netB*	plasmid	toxins, adhesion factors, regulation/ mobilization proteins, enzymes	(NetB): β pore-forming, adhesion, regulating, mobilizing, enzymatic	JF837812	[5]/ [6]
**NELoc-2 11 ORFs**	chromosome	Regulation/ mobilization proteins, enzymes	regulating, mobilizing, enzymatic	JF837813	[5]/ [6]
**NELoc-3 5 ORFs**	plasmid	mobilization proteins, enzymes	mobilizing, enzymatic	NG_041400	[5]/ [6]

Genes investigated for presence among 30 *C. perfringens* isolates. Additionally, NCBI accession number, the genomic localization of the gene, gene product and activity are shown.

References

1. Theoret JR, Uzal FA, McClane BA. Identification and characterization of *Clostridium perfringens* beta toxin variants with differing trypsin sensitivity and in vitro cytotoxicity activity. Infect. Immun. 2015;83:1477–86.

2. Harkness JM, Li J, McClane BA. Identification of a lambda toxin-negative *Clostridium perfringens* strain that processes and activates epsilon prototoxin intracellularly. Anaerobe. Elsevier Ltd; 2012;18:546–52.

3. Miyamoto K, Yumine N, Mimura K, Nagahama M, Li J, McClane BA, et al. Identification of novel *clostridium perfringens* type E strains that carry an iota toxin plasmid with a functional enterotoxin gene. PLoS One. 2011;6.

4. Wade B, Keyburn AL, Seemann T, Rood JI, Moore RJ. Binding of *Clostridium perfringens* to collagen correlates with the ability to cause necrotic enteritis in chickens. Vet. Microbiol. Elsevier B.V.; 2015;180:299–303.

5. Lepp D, Gong J, Songer JG, Boerlin P, Parreira VR, Prescott JF. Identification of accessory genome regions in poultry *clostridium perfringens* isolates carrying the *netB* plasmid. J. Bacteriol. 2013;195:1152–66.

6. Lepp D, Roxas B, Parreira VR, Marri PR, Rosey EL, Gong J, et al. Identification of novel pathogenicity loci in *Clostridium perfringens* strains that cause Avian necrotic enteritis. PLoS One. 2010;5.
