# Supplementary material for: Genome analysis of Clostridium perfringens isolates from healthy and necrotic enteritis infected chickens and turkeys
Source: BMC Res Notes. 2017 Jul 11;10:270. doi: 10.1186/s13104-017-2594-9 (PMC5504799; doi:10.1186/s13104-017-2594-9)
Supplement: Supplementary file 3 — Additional file 3: Table S3. Presence of NELoc-1 and -3 genes in the high prevalence groups. In this pdf-file, information regarding absence/presence of the ORFs that constitute NELoc-1 and -3 can be found. The table includes locus tags and gene product descriptions. [file 13104_2017_2594_MOESM3_ESM.pdf]

Table S3. Presence of NELoc-1 and -3 genes in the high prevalence groups

C24	C26	C31	C33	C36	C37	C41	C48	C124	C125	T11	T53	**NeLoc-1 (33 ORFs)**<br>**Locus tag/gene product**
+	+	+	+	+	+	+	+	+	+	+	+	CP4_3442/transcriptional regulator, MarR family
+	+	+	+	+	+	+	+	+	+	+	+	CP4_3443/β-lactamase domain containing protein
+	+	+		+	+	+		+	+	+	+	CP4_3444/M protein trans-acting positive regulator (MGA)
+	+	+	+	+	+	+	+	+	+	+	+	CP4_3445/putative radical SAM domain containing protein
+	+	+		+	+	+		+	+	+	+	CP4_3446/putative internalin
+	+	+	+	+	+	+	+	+	+	+	+	CP4_3447/putative protein
+	+	+	+	+	+	+	+	+	+	+	+	CP4_3448/putative protein
+	+	+	+	+	+	+	+	+	+	+	+	CP4_3449/NE toxin B (NetB)
+	+	+	+	+	+	+	+	+	+	+	+	CP4_3450/ricin-type β-trefoil domain protein
+	+	+	+	+	+	+	+	+	+	+	+	CP4_3451/transposase for transposon
+	+	+	+	+	+	+	+	+	+	+	+	CP4_3452/hypothetical protein
+	+	+	+	+	+	+	+	+	+	+	+	CP4_3453/hypothetical protein
+	+	+	+	+	+	+	+	+	+	+	+	CP4_3454/chitinase B
+	+	+	+	+	+	+	+	+	+	+	+	CP4_3455/chitodextrinase
+	+	+	+	+	+	+	+	+	+	+	+	CP4_3457/CAAX amino terminal protease
+	+	+	+	+	+	+	+	+	+	+	+	CP4_3458/putative β-toxin
+	+	+	+	+	+	+	+	+	+	+	+	CP4_3459/putative β-toxin
+	+	+	+	+	+	+	+	+	+	+	+	CP4_3460/conserved hypothetical protein
+	+	+	+	+	+	+	+	+	+	+	+	CP4_3461/conserved hypothetical protein
+	+	+	+	+	+	+	+	+	+	+	+	CP4_3462/conserved hypothetical protein
+	+	+	+	+	+	+	+	+	+	+	+	CP4_3464/resolvase recombinase
+	+	+		+	+	+	+	+	+	+	+	CP4_3465/resolvase recombinase
+	+	+	+	+	+	+	+	+	+	+	+	CP4_3466/putative membrane protein
+	+	+	+	+	+	+	+	+	+	+	+	CP4_3468/F5/8 type C domain containing protein
+	+	+	+	+	+	+	+	+	+	+	+	CP4_3469/phosphodiesterase domain 2
+	+	+	+	+	+	+	+	+	+	+	+	CP4_3470/sortase family protein
+	+	+	+	+	+	+	+	+	+	+	+	CP4_3471/putative surface protein
+	+	+	+	+	+	+	+	+	+	+	+	CP4_3472/peptidoglycan bound protein
	+	+	+	+	+	+	+	+	+	+	+	CP4_3473/cell wall surface anchor protein
+	+	+	+	+	+	+	+	+	+	+	+	CP4_3474/signal peptidase I
+	+	+	+	+	+	+	+	+	+	+	+	CP4_3476/hypothetical protein
+	+	+	+	+	+	+	+	+	+	+	+	CP4_3477/swim zinc finger domain
+	+	+		+	+	+	+	+	+	+	+	CP4_3478/diguanylate cyclase
**C24**	**C26**	**C31**	**C33**	**C36**	**C37**	**C41**	**C48**	**C124**	**C125**	**T11**	**T53**	**NeLoc-3** (5 ORFs)<br>Locus tag/gene product
+	+	+	+	+	+	+	+	+	+	+	+	CP4_3567/conserved hypothetical protein
+	+	+		+	+	+	+		+	+	+	CP4_3568/hypothetical protein
+	+	+	+	+	+	+	+	+	+	+	+	CP4_3569/resolvase/recombinase
+	+	+	+	+	+	+	+	+	+	+	+	CP4_3570/conserved hypothetical protein
+	+	+	+	+	+	+	+	+	+	+	+	CP4_3572/NADP-dependent 7-α-HSD

The locus tag and the gene product of the NELoc-1 and -3 associated genes are shown (Lepp et al., 2013). + indicates presence respectively, among the *C. perfringens* isolates. Only isolates with high prevalence of the NELoc-1 and -3 genes are presented.
