# Supplementary material for: Genome analysis of Clostridium perfringens isolates from healthy and necrotic enteritis infected chickens and turkeys
Source: BMC Res Notes. 2017 Jul 11;10:270. doi: 10.1186/s13104-017-2594-9 (PMC5504799; doi:10.1186/s13104-017-2594-9)
Supplement: Supplementary file 4 — Additional file 4: Table S4. NELoc-2 genes identified among the C. perfringens isolates. In this pdf-file, information regarding absence/presence of the ORFs that constitute NELoc-2 can be found. The table includes locus tags and gene product descriptions. [file 13104_2017_2594_MOESM4_ESM.pdf]

Table S4. NELoc-2 genes identified among the *C. perfringens* isolates

C7	C8	C24	C26	C31	C33	C36	C37	C41	C48	C124	C125	T1	T5	T11	T43	**NeLoc-2 (11 ORFs) Locus tag/gene product**
+	+	+	+	+	+	+	+	+	+	+	+	+	+	+	+	CP4_0458/sigma factor SgiI
+	+	+	+	+	+	+	+	+	+	+	+	+	+	+	+	CP4_0459/conserved hypothetical protein
+	+	+	+	+	+	+	+	+	+	+	+	+	+	+	+	CP4_0460/putative VTC domain superfamily
+	+	+	+	+	+	+	+	+	+	+	+	+	+	+	+	CP4_0461/ putative tubulin/FtsZ, GTPase
+	+	+	+	+	+	+	+	+	+	+	+	+	+	+	+	CP4_0462/resolvase
+	+	+	+	+	+	+	+	+	+	+	+	+	+	+	+	CP4_0463/putative VTC domain superfamily
+	+	+	+	+	+	+	+	+	+	+	+	+	+	+	+	CP4_0464/ tubulin/FtsZ, GTPase
+	+	+	+	+	+	+	+	+	+	+	+	+	+	+	+	CP4_0465/ CotH protein
+	+	+	+	+	+	+	+	+	+	+	+	+	+	+	+	CP4_0466/conserved hypothetical protein
+	+	+	+	+	+	+	+	+	+	+	+	+	+	+	+	CP4_0467/putative heat repeat
+	+	+	+	+	+	+	+	+	+	+	+	+	+	+	+	CP4_0468 chitin synthase

The locus tag and the gene product of the NELoc-2 associated genes are shown (Lepp et al., 2013). + indicates presence of genes and NELoc-2 was fully conserved among all isolates from this table.
