# Supplementary material for: Genome analysis of Clostridium perfringens isolates from healthy and necrotic enteritis infected chickens and turkeys
Source: BMC Res Notes. 2017 Jul 11;10:270. doi: 10.1186/s13104-017-2594-9 (PMC5504799; doi:10.1186/s13104-017-2594-9)
Supplement: Supplementary file 5 — Additional file 5. NT and AA sequence of the putative collagen adhesion gene cnaD. [file 13104_2017_2594_MOESM5_ESM.pdf]

cnaD(2787NTs)

ATGAATAGTAAGGGTATAGATACCAAAAAATTAATGTCAATTATAAGCTTAATAATGACAGTTATATTTTTAAGTATTTTAT
TGCCTACTAATTTAACAAAAGCAGAAGAAAAGTCTGATAGCATGTCTGTTGAAAAAGTATTAAATTCAGAAATGTATAATA
ATTTCAATAATGAAATATTAAACAATAAAAGTAGTATTAATTACACTACTGATAATAATCAAGGTACATGGCCAGTAAATTG
GGAGTATGGAAATGTAAGCAATAAAAATAAAATAAACAAAAGTGTATATGGCAAGAATCAACCATTAGAATATAATGAAG
GATATTTAACAAAGAAAGCTTATACAACAGATGAAGATAATGTATTTGACATTAATCTAAAGATTCAGGGGAAAAAAAATC
AATCTTTAAAAAAAGATGTGGTTTTCCTTTTAGACAATTCAAATTCTATGACAACAAATAATCGTGCAATAAAGATTAAAGA
ACAAATTAAAAATGTTATGGATAAGCTAAATACTAATAATACACGTTATGCGCTTGTTACTTATGCCTCAACAATTTTAGAT
GGAAGGTATTATCATTTAATTGATAGATCTATAGGGGATAATAAATATACAGTTTATAAAGGTTATACAAGTAACCAGTGT
TATCTAAATTTTACTAGTAATATTCAAGAGATTTATAATAAAATACCTACTACTGTTCCAAATCAGAGAAATAATGGTTATGT
AGGGGGAACATTTACTCAAGAAGGATTATTGAAAGCAATAGAACTTTTAAAAAAATAGTGATGCTGATGAAAAAATTATTA
TTCATCTTACTGATGGGTTACCAACATTTTCTTTTCTTTTAAAAGAGTTTGGAGGAAATGAAAAAGCTATTTTTGACTATAAC
ACTCAATATAATGGTATTGGTGTACGTGGATTTGGGACATCATACTTTTTTAATACTAAAACTCAAAAGCCGTATATATATT
CTAGAGAAGAAGTATATTCTGCTTTAAATCGTTCAATAAATAAAAATGAATCAATATGGAATAATGGTTTTCCAACTACTTT
AGAAGCAGAGAATATTAAAAAGGAAAATCCGGACATTAATATTTACACTATTGGGATAGAACTTAAAAAAGAAGTATATA
AATGGGATGATTATAGAAAATATTATAATGCTGAAGGTGTTGTTGAACTTCCAGAAATAAAAAAATTCTTAGAATCAATTT
CTTCTAGTCCTGCTGAGGCTTTTGTTAATGAAAATGTTGATGATATTGATGAGATTATTAATAAAATTATTGATAAAATAAA
GAATTCAATAAATGATGGTACTGTTATAGATCCTATGGGTGATATGGTTTATATTGTTAAAAATGGAGAATTTAATAATGA
GGATTATAAGTTAACGGCATCTAATAATAAGTTATTAGAAGGTGTAAAAGTAGGATATAACGAAAAAAATAGACAGATAG
TTCTTACAGGACTAAATTTAGGTGAAAATGAATGGGTTGAATTAAATTATAAAGTAAGATTAAATACAAGCAACCCTGACT
TTAATGGGGATTTTTGGTATCAAGCTAATAAAAGAACTGTTTTAAACCCTAATAATAAAGAACCAAATATTTTTCGTGACTT
TGTAATACCTTCTGTTAGTGGAAAAAGACCATCAATAGAAGTTAAATTAAAGAAGATATCAAGTGAAACATCAAAGCCATT
AGCTAATTCAGAATTTGAACTTTATAATTCTATAAATGAAAAATTAGGCTCTTTTACAACAAAAGAAAATGGGGAGGTAAG
TCTTGGGTATTTACCAGAAGGTGAGTATAAGTTAAAAGAAATAACCCCACCAAAAGGATATATTTTATCTAAAGACTTTATT
AATTTTAAGATTAATAATGGAAAGGCTATACAAGATGGAAATGAAGTAGAATTTATTACGGTAAGTAATAAAGTTAATAGT
ATATGTATAAAGAAAACTGATGATGCAAAGTTAGAAGCTGATGCTAAATTTTTAAGTGGAGCAAAATTTGAATTAAATAAA
GCTAATGATAAAAATTTCAAACCTTTAGTAAAAGAAACTGATGATAAGGGAGAAATAGAATTTAATGAGATTGAACCTGG
TACTTACTATTTAAAAGAAGTTCTTGCTCCTAATGGATATGAACAAATTAAGGAAGACATAGGACCAATTGTAGTTGATAA
TACTGGTGTAGTAACAATTCCTTGGGATAAATTAAAAAGCAACGATGTAGAAAAGTGGAATAATCAAGAGATTATTCGTAT
AAAAAATAAAAAGTTGAAATCGGCTGTATACATAGATAAAGTAGATGCAATAAATCAAGGAATAAAGTTAAGTGGAGCAA
AATTTTCACTTTATACTAATGATGAAAATTATAAAAATGATAAAAAGCTAGTAAGAAATGGTGTAAATTATTATTTAATAAG
TGAAAAAGTATCAAATTATGAAGGTAGAATTGAATGGGATAATCTTAATTCAGGACAAGAATATAAATATCTTATTCAGGA
GACTGAGGCACCAAAGGGATATACTGTAAGTGGAAAAGAAATATTATTCCATTTTAAAGATAATACTGTTGTTATAGATAA
TGAGAATGATGTAAAAGCACTTGCTAGTATAAATGGACAAGTTATTAGTATTAAAAATGCTAAAATATATAAGCTTCCGTC
ATCTGGCGGTATCGGAGTGTACCCTTTCTTACTTATAGGGACACTATGTATGGCTTTAAGTTTAATATATAGTTTTAATAGT
AAGGTTTTAAATAAAAGGAAATAA

cnaD(928AAs)

MNSKGIDTKKLMSIISLIMTVIFLSILLPTNLTKAEEKSDSMSVEKVLNSEMYNNFNNEILNNKSSINYTTDNNQGTWPVNWEYG
NVSNKNKINKSVYGKNQPLEYNEGYLTKKAYTTDEDNVFDINLKIQGKKNQSLKKDVVFLLDNSNSMTTNNRAIKIKEQIKNVM
DKLNTNNTRYALVTYASTILDGRYYHLIDRSIGDNKYTVYKGYTSNQCYLNFTSNIQEIYNKIPTTVPNQRNNGYVGGTFTQEGLL
KAIELLKNSDADEKIIIHLTDGLPTFSFLLKEFGGNEKAIFDYNTQYNGIGVRGFGTSYFFNTKTQKPYIYSREEVYSALNRSINKNESI
WNNGFPTTLEAENIKKENPDINIYTIGIELKKEVYKWDDYRKYYNAEGVVELPEIKKFLESISSSPAEAFVNENVDDIDEIINKIIDKIK
NSINDGTVIDPMGDMVYIVKNGEFNNEDYKLTASNNKLLEGVKVGYNEKNRQIVLTGLNLGENEWVELNYKVRLNTSNPDFN
GDFWYQANKRTVLNPNNKEPNIFRDFVIPSVSGKRPSIEVKLKKISSETSKPLANSEFELYNSINEKLGSFTTKENGEVSLGYLPEGE
YKLKEITPPKGYILSKDFINFKINNGKAIQDGNEVEFITVSNKVNSICIKKTDDAKLEADAKFLSGAKFELNKANDKNFKPLVKETDD
KGEIEFNEIEPGTYYLKEVLAPNGYEQIKEDIGPIVVDNTGVVTIPWDKLKSNDVEKWNNQEIIRIKNKKLKSAVYIDKVDAINQGI
KLSGAKFSLYTNDENYKNDKKLVRNGVNYYLISEKVSNYEGRIEWDNLNSGQEYKYLIQETEAPKGYTVSGKEILFHFKDNTVVID
NENDVKALASINGQVISIKNAKIYKLPSSGGIGVYPFLLIGTLCMALSLIYSFNSKVLNKRK*
